# Cyclic AMP Accumulation in Migraine Induction

**DOI:** 10.15844/pedneurbriefs-29-1-6

**Published:** 2015-01

**Authors:** Ana B. Chelse, Leon G. Epstein

**Affiliations:** 1 Division of Neurology, Ann & Robert H. Lurie Children's Hospital of Chicago, Chicago, IL; 2 Departments of Pediatrics and Neurology, Northwestern University Feinberg School of Medicine, Chicago, IL

**Keywords:** Migraine, Headache, Cyclic adenosine monophosphate

## Abstract

Investigators from Danish Headache Centre, Glostrup Hospital, and the University of Copenhagen investigated whether intracellular cyclic AMP accumulation induced migraine attacks among 14 migraine patients without aura.

Investigators from Danish Headache Centre, Glostrup Hospital, and the University of Copenhagen investigated whether intracellular cyclic AMP accumulation induced migraine attacks among 14 migraine patients without aura. Patients either received placebo or cilostazol, a selective inhibitor of phosphodeisterase type 3 (PDE3) which prohibits the breakdown of cyclic AMP (cAMP). Patients were instructed to record their headache symptoms, localization and characteristics by a self-administered questionnaire every hour until 13h post-administration. Twelve of 14 patients (86%) developed migraine-like attacks after cilostazol compared with two patients (14%) after placebo. The median time to migraine onset was 6h post cilostazol (range 3-11). Eleven of 14 (79%) took rescue medication cilostazol and 2 (14%) after placebo (*P* = 0.003). The headaches induced were similar to their usual migraines and were effectively aborted by their usual migraine treatment. Nausea was significantly different between the two groups (P = 0.027). Photo- and phonophobia were not significantly different between groups. The authors suggest that the intracellular accumulation of cyclic AMP induces migraine attacks, which furthers our understanding of the migraine pathophysiology. [1]

COMMENTARY. The initiating mechanisms of migraine attacks are complex and not fully understood. Human experimental migraine models have led to increased knowledge in understanding the migraine pathway. Nitric oxide (NO) has been well established to cause migraines. Studies in healthy volunteers and migraine patients with sildenafil, a highly selective inhibitor of phosphodiesterase type 5 (PDE5) showed that cyclic guanosine monophosphate (cGMP) and cAMP are likely mediator of headache responses elicited by NO [2]. Calcitonin gene-related peptide (CGRP), an endogenous neuropeptide, has also been shown to play a role in migraine attack [3]. CGRP's vascular effects are also mediated by an increase in cAMP. Prior research showed that PDE3A and PDE5A are each present in the trigeminal ganglion of rats [4]. This work also suggested a link between the cAMP and cGMP pathways in migraine induction. Sildenafil and cilostazol were shown to cause an inhibition of cAMP hydrolysis in the rat trigeminal ganglion through inhibition of PDE3 [4]. The current study added to this knowledge by showing that cilostazol causes an accumulation of cAMP in humans and induces migraine attacks. Activation of cAMP-selective phosphodeisterases (PDE3 or PDE5) should be considered as a possible new target in migraine treatment.
